# Temporal Trends in Outcomes and Predictors of Length of Stay Following Lung Cancer Resection Over 10 Years With Enhanced Recovery After Surgery

**DOI:** 10.1093/icvts/ivaf216

**Published:** 2025-10-17

**Authors:** Lauren Kari Dixon, David Messenger, Lesley Wood, Neil Rasburn, Douglas West, Eveline Internullo, Rakesh Krishnadas, Igor Saftic, Stylianos Gaitanakis, Laura Socci, Michelle Brack, Timothy Batchelor, Natasha Joshi

**Affiliations:** Department of Thoracic Surgery, University Hospitals Bristol and Weston, Bristol, BS28HW, United Kingdom; Department of Health Services Research and Policy, London School of Hygiene & Tropical Medicine, London, WC1E 7HT, United Kingdom; Department of Colorectal Surgery, University Hospitals Bristol and Weston, Bristol, BS28HW, United Kingdom; University Hospitals Bristol and Weston, Bristol, BS28HW, United Kingdom; Department of Anaesthesia, University Hospitals Bristol and Weston, Bristol, BS28HW, United Kingdom; Department of Thoracic Surgery, University Hospitals Bristol and Weston, Bristol, BS28HW, United Kingdom; Department of Thoracic Surgery, University Hospitals Bristol and Weston, Bristol, BS28HW, United Kingdom; Department of Thoracic Surgery, University Hospitals Bristol and Weston, Bristol, BS28HW, United Kingdom; Department of Thoracic Surgery, University Hospitals Bristol and Weston, Bristol, BS28HW, United Kingdom; Department of Thoracic Surgery, University Hospitals Bristol and Weston, Bristol, BS28HW, United Kingdom; Department of Thoracic Surgery, University Hospitals Bristol and Weston, Bristol, BS28HW, United Kingdom; ERAS Specialist Nurse, University Hospitals Bristol and Weston, Bristol, BS28HW, United Kingdom; Department of Thoracic Surgery, St Bartholomew’s Hospital, London, EC1A 7BE, United Kingdom; Department of Anaesthesia, University Hospitals Bristol and Weston, Bristol, BS28HW, United Kingdom

**Keywords:** ERAS, lung cancer, thoracic surgery, lobectomy, VATS

## Abstract

**Objectives:**

Enhanced Recovery After Surgery aims to accelerate recovery, with length of stay as a key metric. This study assessed temporal trends in short-term outcomes within a maturing programme and identified factors associated with increased hospital stay.

**Methods:**

Data were prospectively collected for consecutive patients undergoing lung cancer resection following a 14-step protocol between 2013 and 2023. Primary outcome was length of stay. Secondary outcomes included 30-day mortality, morbidity, re-admission, and reoperation rates. Predictors of length of stay were analysed using linear regression.

**Results:**

We included 2192 patients; procedures included lobectomy (61%), wedge resection (23%), segmentectomy (10%), pneumonectomy (3.5%), and bi-lobectomy (2.7%). Video-assisted thoracoscopic surgery was used in 80% of cases. Median length of stay decreased from 5 to 4 days (*P* < .001), while protocol adherence increased from 10/14 to 12/14 (*P* = .01). In-hospital mortality (2.9% to 1.0%, *P* < .001) and major morbidity (12.2% to 5.6%, *P* < .001) both declined. In multivariable linear regression, factors associated with longer stay included age (β = 0.17, CI 0.13 to 0.20, *P* < .001), higher American Society of Anesthesiologists score (β = 1.12, CI 1.04 to 2.2, *P* = .02), open surgery (β = 1.0, CI 0.17 to 2.2, *P* = .043), thoracoscopic-to-open converted surgery (β = 1.49, CI 0.96 to 1.9, *P* = .03), and intensive care (β = 3.4, CI 2.5 to 4.3, *P* < .001). Protective factors were early mobilization (β = −0.90, CI −1.9 to 0.33, *P* = .005) and opioid avoidance (β = −0.72, CI −2.4 to 0.99, *P* = .038).

**Conclusions:**

Sustained use of an Enhanced Recovery After Surgery programme was associated with shorter hospitalization and reduced morbidity. Factors associated with length of stay can identify patients at risk of delayed recovery and prioritize elements for optimization within recovery pathways.

## Graphical abstract

**Figure:**
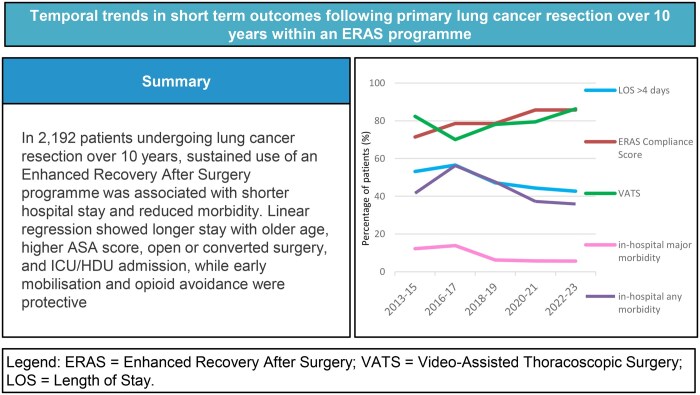

## INTRODUCTION

Enhanced Recovery After Surgery (ERAS) protocols are evidence-based, multimodal peri-operative pathways designed to accelerate recovery, reduce hospital stay, and improve outcomes.^1^^,^^2^ Initially developed in colorectal surgery,^3^ ERAS protocols have been successfully implemented in many specialities, including thoracic surgery.^4^^,^^5^

Lung cancer is the third most common cancer in the United Kingdom.^6^ For early-stage non-small cell lung cancer, anatomical resection is the standard treatment.^7^ According to a 2020 survey, 34 out of 36 UK thoracic surgery centres use ERAS pathways.^8^ Prior studies have shown improved patient outcomes with higher ERAS adherence.^9^^,^^10^ The ERAS protocol used in our centre is comprised of 14 key elements (**Table 1**).

**Table 1.** The ERAS Protocol at Our Thoracic Surgery Centre

Pre-operative	1	Pre-operative visit
	2	Pre-operative assessment
	3	Patient education of ERAS
	4	Admission on the day of surgery
	5	Pre-operative carbohydrate drink
Peri-operative	6	Regional Anaesthesia
	7	Prophylactic antibiotics at induction
	8	Avoidance of sedative preanaesthetic medication
	9	Intraoperative warming
Post-operative	10	Avoidance of post-operative intravenous fluids
	11	Avoidance of opioid analgesia
	12	Early return to feeding
	13	Targeted post-operative nausea and vomiting therapy
	14	Mobilization within 24 hours

Abbreviation: ERAS, Enhanced Recovery After Surgery.

Despite widespread adoption, the long-term sustainability of ERAS within thoracic surgery is underexplored. In colorectal surgery, compliance has declined over time, potentially undermining initial benefits.^11^

Reducing length of stay (LOS) is a central aim of ERAS, offering clinical and economic value.^11^ While concerns have been raised regarding premature, re-admission rates have not risen in modern thoracic ERAS programmes,^2^ and LOS remains a core outcome when evaluating peri-operative care.^12^

The aim of this study was to examine temporal trends in short-term outcomes within a maturing thoracic ERAS programme and to identify factors associated with LOS.

## METHODS

This study followed STROBE reporting guidelines^13^ and was approved by the local audit department (reference: THOR/SE/2024-25/05). Data were used in compliance with the World Medical Association Declaration of Taipei. The requirement for patient consent was waived as only routinely collected, anonymized audit data were analysed. Data are available from the corresponding author upon reasonable request.

### Patients

All consecutive patients undergoing primary lung cancer resection between August 2013 and July 2023 in a thoracic surgery department were included. Patients under 18 years of age and patients with benign or metastatic disease were excluded.

### Patient experience

At pre-operative assessment, patients were given an ERAS diary with daily targets. This visit also addressed comorbidities and organized relevant investigations (eg, pulmonary function tests, echocardiography, cardiopulmonary exercise testing as indicated). High-risk patients were discussed at a multidisciplinary complex case meeting to plan optimization and post-operative care.

Patients were admitted on the day of surgery and given a carbohydrate drink 2 hours before anaesthesia. At induction, patients received prophylactic antibiotics and regional anaesthesia (paravertebral catheter and intercostal blocks with levobupivacaine). Intraoperative care included warming and euvolemic fluid management. Video-assisted thoracoscopic surgery (VATS) was the standard approach. Most patients were admitted to the thoracic ward post-operatively; high-risk patients and all pneumonectomy cases were admitted to the high-dependency unit (HDU) or intensive care unit (ICU) electively.

Post-operative oral intake resumed early. Paravertebral analgesia was continued for up to 72 hours, with regular paracetamol and non-steroidal anti-inflammatory drugs; opioids were used as required. Gabapentinoids were not used routinely, but were prescribed selectively. Patients were mobilized with physiotherapy within 24 hours and reviewed daily. Chest drains were removed with the absence of air leak for 6 hours and output <500 mL over 24 hours.^14^

### Data and outcomes

Data were routinely collected by the ERAS data manager, supplemented by case note review. Case notes were reviewed in detail, and where no documentation of an intervention was available, it was assumed not to have been delivered, minimizing missingness. Data included demographics, diagnosis, operative details, and adherence to ERAS elements. Adherence was recorded against the ERAS elements and scored out of 14.

The primary outcome was LOS, analysed as a continuous variable. To approximate recovery independent of complications, primary regression models included only patients without post-operative morbidity. Secondary analysis, including all patients, was subsequently performed. A binary outcome of prolonged LOS (>4 days), corresponding to the cohort median and published averages,^15^ was evaluated as a secondary outcome. Other secondary outcomes included 30-day mortality, in-hospital morbidity (using a thoracic-modified Clavien-Dindo classification^16^), 30-day re-admission, reoperation, and ERAS adherence. Major morbidity was defined as Clavien-Dindo classification of 3/4.

### Statistical analysis

Categorical variables are presented as frequencies and percentages; continuous variables as medians with IQRs. Descriptive comparisons between groups used chi-squared tests for categorical variables and Mann-Whitney *U* tests for continuous variables. Temporal trends were evaluated using chi-squared tests for categorical variables and Kruskal-Wallis tests for continuous variables. To assess ordered trends across year groups, Cochran-Armitage trend tests were applied for categorical variables, and the Jonckheere-Terpstra test was used to confirm monotonic trends in adherence scores. Adherence scores were also modelled using linear regression with year as an ordinal variable.

Descriptive comparisons of patient groups (≤4 vs >4 days) are presented in tables, but linear regression modelling provides the basis for inference. Multivariable models included pre-operative, intraoperative, and peri-operative ERAS process measures to identify factors associated with LOS. These were not intended as pre-operative prediction models but to evaluate associations with outcomes, as some ERAS elements are modifiable peri-operatively and may identify patients at risk in real time.

Variables with *P* < .1 in univariable analysis were considered for entry into multivariable models, which were selected using the Akaike information criterion. Analyses used complete-case data. Odds ratios (ORs) or β coefficients with 95% CIs and *P*-values are reported. Alpha error was set at 0.05 (2-tailed). Statistical analyses were performed using R version 1.4.1106.^17^

## RESULTS

From 2013 to 2023, 2192 consecutive patients underwent lung cancer resection. The proportion of each procedure was: 60.5% lobectomy, 23.3% wedge resection, 10.0% segmentectomy, 3.5% pneumonectomy, and 2.7% bi-lobectomy. A VATS approach was used in 80% of cases, 14% via thoracotomy (open), and 6% were VATS-to-open conversions.

### Outcomes over time

Trends in approach, ERAS adherence, and outcomes are summarized in **Table 2a**. While median age was stable (*P* = .5), American Society of Anesthesiologists (ASA) grade ≥3 increased from 38.0% in 2013-2015 to 56.5% in 2022-2023 (*P* = .02). VATS usage increased from 82.3% to 86.3% (*P* < .001), and planned HDU/ICU admissions decreased from 26.1% to 19.3% (*P* < .001).

**Table 2.** Trends in Operations, Approach, and Outcomes Over the 10-Year Study Period: (a) Including All Patients and (b) Including Only Patients Without Post-Operative Morbidity

(a) All patients	2013-2015	2016-2017	2018-2019	2020-2021	2022-2023	*P*-value^b^
	*N* = 534^a^	*N* = 398^a^	*N* = 485^a^	*N* = 386^a^	*N* = 389^a^	
Age (years)	70 (61-75)	69 (61-75)	72 (60-76)	71 (62-76)	72 (63-76)	.5
ASA (%)						.02
1	1.9	1.3	0.5	1.3	0.5	
2	60.1	47.7	45.8	42.2	41.2	
3	38.0	50.5	55.4	56.2	57.3	
4	0	0.5	0.3	0.3	1.0	
Approach (%)						<.001
VATS	82.3	70.1	78.1	79.4	86.3	
Open	12.3	24.6	18.7	10.1	6.9	
Converted	5.2	5.3	4.1	9.8	6.7	
Planned ICU/HDU admission (%)	26.1	24.5	22.2	20.5	19.3	<.001
LOS (days)	5 (3-7)	5 (3-8)	4 (3-6)	4 (3-6)	4.0 (3-6)	<.001
Prolonged LOS (%)	53.1	56.5	47.1	44.3	42.7	<.001
Adherence score (out of 14)	10 (8-12)	11 (10-12)	11 (10-12)	12 (11-12)	12 (11-13)	.01
30-day mortality (%)	2.9	3.2	1.2	1.0	1.0	<.001
30-day re-admission (%)	5.1	6.5	7.4	8.5	6.9	.3
In-hospital any morbidity (%)	41.6	56.2	47.6	37.3	35.9	<.001
In-hospital major morbidity (%)	12.2	13.8	6.2	5.7	5.6	<.001

(b) Any morbidity excluded	2013-2015	2016-2017	2018-2019	2020-2021	2022-2023	*P*-value^b^
	*N* = 308^a^	*N* = 174^a^	*N* = 254^a^	*N* = 242^a^	*N* = 243^a^	
Age (years)	70 (60-73)	70 (60-75)	72 (60-77)	70 (62-76)	73 (63-76)	.6
ASA (%)						.04
1	1.9	1.1	0.3	1.2	0.4	
2	59.1	49.7	48	44	40	
3	39.0	49	52	55	58	
4	0	0.6	0.5	0	0.8	
Approach (%)						<.001
VATS	81.3	72.1	80.1	81.4	88.3	
Open	12.3	24.6	16.7	10.7	6.6	
Converted	6.4	3.3	3.2	7.9	5.1	
Planned ICU/HDU admission (%)	13.1	10.5	11.2	7.5	6.3	<.001
LOS (days)	5 (3-7)	5 (3-8)	4 (3-6)	4 (3-6)	4 (3-6)	<.001
Prolonged LOS (%)	54.2	51.1	42.9	41.7	41.2	.009
Adherence score (out of 14)	12 (9-13)	12 (11-12)	12 (11-12)	12 (11-13)	12 (11-13)	.01
30-day mortality (%)	2.9	3.2	1.2	1.0	1.0	<.001
30-day re-admission (%)	6.2	7.5	8.7	2.9	2.5	<.001

a *n* (%); median (IQR).

b Pearson’s chi-squared test; Kruskal-Wallis rank sum test.

Abbreviations: ASA, American Society of Anesthesiologists; HDU, high-dependency unit; ICU, intensive care unit; LOS, length of stay; VATS, video-assisted thoracoscopic surgery.

Median LOS reduced from 5 days (IQR 3-7) to 4 days (IQR 3-6) (*P* < .001). Thirty-day mortality fell from 2.9% to 1.0% (*P* < .001), and major morbidity from 12.2% to 5.6% (*P* < .001). Re-admission rates remained consistent (*P* = .3). Findings were consistent when categorical outcomes were assessed with Cochran-Armitage trend tests, confirming monotonic temporal trends.

Median ERAS adherence was 12 and showed improvement from 10 (IQR 8-12) to 12 (IQR 11-13) (*P* = .01) (see **Figure S1**). Per-element adherence over time highlights consistently high adherence for elements such as prophylactic antibiotics and regional anaesthesia, while opioid avoidance and early mobilization showed persistently lower adherence compared with other items (**Table S2**). In a linear regression model (all patients), adherence scores increased significantly over time (β = 0.20, CI 0.17-0.23, *P* < .001), supported by the Jonckheere-Terpstra test (JT = 666 588; *P* < .001).

Among the 1210 patients without post-operative morbidity (**Table 2b**), trends were similar, but 30-day re-admissions also improved from 6.2% to 2.5% (*P* < .001).

### Short-term outcomes

Baseline characteristics, operative details, and ERAS adherence are summarized in **Table S1**. **Table 3** summarizes post-operative outcomes. Median LOS was 4 days. Re-admissions, re-operations, and morbidity were all more frequent among patients with prolonged LOS.

**Table 3.** Post-Operative Outcomes

Characteristic	All, *N* = 2192^a^	Normal LOS, *N* = 1122^a^	Prolonged LOS, *N* = 1070^a^	*P*-value^b^
Length of stay	4 (3, 7)	3 (3, 4)	7 (5, 10)	<.001
30-day mortality	27 (1.2%)	8 (0.7%)	18 (1.7%)	.067
30-day re-admission	149 (6.8%)	38 (3.4%)	79 (7.4%)	<.001
30-day reoperation	102 (4.7%)	8 (0.7%)	94 (8.8%)	<.001
In-hospital any morbidity	982 (44.7%)	281 (25%)	701 (65.5%)	<.001

a Median (IQR); *n* (%).

b Pearson’s chi-squared test.

Abbreviation: LOS, length of stay.

### Factors associated with LOS

Among patients without post-operative morbidity (*n* = 1210), multivariable linear regression identified factors associated with longer LOS: older age, ASA, open surgery, VATS-to-open conversion, and ICU/HDU admission. Protective factors included early mobilization and opioid avoidance (**Table 4**).

**Table 4.** Factors Associated With Length of Stay (Continuous Outcome, Patients Without Morbidity)

	Univariable analysis			Multivariable analysis		
	β Coefficient^a^	95% CI	*P*-value	β Coefficient^a^	95% CI	*P*-value
Age	0.52	0.18, 0.83	.02	0.17	0.13, 0.20	<.001
Male sex	−0.65	−0.95, 0.16	.12			
ASA	1.07	0.96, 1.27	.03	1.12	1.04, 2.2	.02
Year of surgery group	−0.27	−0.59, 0.04	.086	−0.31	−0.59, −0.04	.027
Procedure						
Lobectomy	—	—				
Bilobectomy	0.97	−2.1, 4.0	.5	0.90	0.3, 1.5	.5
Pneumonectomy	7.0	4.4, 9.7	<.001	0.05	0.2, 2.3	.7
Segmentectomy	−1.9	−3.5, −0.36	.015	−1.1	−2.3, 0.10	.073
Wedge	−1.3	−2.4, −0.25	.016	−0.75	−1.6, −0.14	.04
Approach						
VATS	—	—				
Open	3.5	2.1, 4.8	<.001	1.0	0.17, 2.2	.043
Converted	4.5	2.58, 5.4	<.001	1.49	0.96, 1.9	.03
Pre-operative creatinine	0.00	−0.01, 0.02	.7			
Pre-operative haemoglobin	0.90	0.83, 0.97	.007	−0.01	−0.02, 0.00	.095
Explanation of ERAS	−0.9	−2.4, 0.27	.2			
Day of surgery admission	−2.8	−9.0, 3.4	.4			
Pre-operative carbohydrate drink	−0.21	−0.98, −0.05	.03	−0.35	−0.35, 0.1	.05
Avoid sedation	−1.4	−7.9, 2.8	.13			
Prophylactic antibiotics	−1.4	−3.3, 0.58	.2			
Regional anaesthesia	0.5	−0.35, 2.4	.3			
Warming	−0.52	−4.4, 5.5	.8			
Avoidance of IV fluids	−0.24	−9.2, 9.7	>.9			
Avoidance of opioids	−0.93	−3.2, −0.1	.05	−0.72	−2.4, −0.99	.038
Early returning to oral feeding	−0.41	−1.7, 0.90	.5			
Targeted nausea and vomiting therapy	−0.57	−3.2, 2.1	.7			
Early mobilization	−0.56	−1.5, −0.39	.02	−0.9	−1.9, −0.33	.005
HDU/ICU admission	4.9	3.9, 5.9	<.001	3.4	2.5, 4.3	<.001
Surgeon identifier^b^			.13			

a β = regression coefficient from linear model. Positive values indicate longer stay; negative values indicate shorter stay.

b Categorical variable with 7 levels. *P*-value from likelihood ratio test comparing the model with and without surgeon identifier.

Abbreviations: ASA, American Society of Anesthesiologists; ERAS, Enhanced Recovery After Surgery; HDU, high-dependency unit; ICU, intensive care unit; LOS, length of stay; VATS, video-assisted thoracoscopic surgery.

When analysed as a binary outcome where prolonged LOS >4 days, factors associated with prolonged LOS included older age, higher ASA score, open surgery, VATS-to-open conversion, and ICU/HDU admission (**Table S4**). Protective factors included early mobilization, pre-operative carbohydrate drinks, post-operative opioid avoidance, and wedge resection. Surgeon identifier was not associated with LOS. Findings were similar when all patients were included (**Tables S3 and S5**).

### Predictors of in-hospital morbidity

Predictors of in-hospital morbidity included age, pneumonectomy, VATS-to-open conversion, and ICU/HDU admission. Protective factors were surgery during 2022-2023 (vs 2013-15) and early mobilization (**Table S5**).

## DISCUSSION

Our study describes the sustained implementation of the ERAS programme over 10 years and its association with improved short-term outcomes after lung cancer resection. Protocol adherence increased over time (with reduced inter-patient variation), accompanied by reductions in LOS, morbidity, and mortality, demonstrating that ERAS is safe, effective, and achievable by patients.

While LOS is sometimes criticized as a metric, it remains a widely accepted measure of ERAS success.^12^ Factors independently associated with increased LOS included older age, open/converted surgery, HDU/ICU admission, while early mobilization and opioid avoidance were protective. Importantly, ERAS protocol adherence was associated with improved outcomes, likely reflecting both the impact of key care elements and the compound benefit of multiple small improvements working synergistically.^18^ These findings are consistent with earlier work at our centre, which also demonstrated improved ERAS adherence and reduced LOS.^9^

ERAS pathway elements were included in the analysis in the present study alongside pre- and intraoperative factors. These were not intended for pre-operative risk prediction but rather as peri-operative process measures. Identifying their association with LOS is valuable because they are modifiable during the peri-operative period and may help clinicians recognize patients at risk of delayed recovery in real time.

Our findings support those of the VIOLET trial, where VATS reduced LOS by 1 day compared to open surgery.^19^ In our cohort, reduced LOS was not associated with increased re-admissions, easing concerns about premature discharge, aligning with other studies showing ERAS programmes reduce LOS without compromising re-admission rates.^20^ One study reported similar LOS between open and VATS approaches within ERAS^21^; our finding of longer LOS in open and converted cases may reflect increased complexity or intraoperative complications. Although reasons for conversion were not captured in our dataset, conversions most often occur due to bleeding, adhesions, or tumour/anatomical constraints^22^; these factors likely contributed in our series. The study period coincided with increasing use of VATS, with surgeons reaching the peak of their learning curve. The higher conversion rate of later years may reflect growing confidence in attempting complex cases minimally invasively. Surgeon identity was not significantly associated with LOS, suggesting improvements were driven through effective multidisciplinary coordination.

Early mobilization was protective against increasing LOS; it can prevent complications such as venous thromboembolism, pneumonia, and muscle wasting.^4^^,^^23^ Older age and ICU/HDU admission were associated with increasing LOS. While some factors are non-modifiable, risk may be mitigated through pre-operative optimization, adherence to key ERAS elements, and prioritizing VATS. Early identification of high-risk patients enables targeted interventions.

Median LOS plateaued from 2018 onwards, likely reflecting maturation of the ERAS programme. Further improvements could be made by targeting ERAS elements of poor adherence, especially opioid avoidance. Post-operative pain is a cause of prolonged hospital stay after VATS,^24^ and opioid side effects, such as nausea, constipation, and confusion, can further delay recovery.^23^ While regional anaesthesia was routinely used, multimodal non-opioid analgesia regimes reduce pain and opioid requirements.^25^ Additionally, early chest drain removal in appropriate patients can reduce pain and opioid use, facilitating enhanced recovery without compromising patient safety.^18^^,^^23^^,^^26^

The sustained use of ERAS over a decade in our centre contrasts with other examples, where maintaining long-term adherence has been challenging.^27^ Continued clinical engagement, multidisciplinary teamwork, and audit with feedback ensured our ERAS protocol was embedded within a culture of continuous quality improvement to sustain long-term benefits.^28^

Robotic-assisted thoracic surgery (RATS) was not implemented at our centre during the study period. Recent studies have shown that RATS offers comparable short-term outcomes to VATS.^29–31^ Although the integration of RATS within a mature ERAS programme remains underexplored, prior evidence suggests ERAS protocols can be effective in robotic surgery.^25^ Future research should assess how RATS influences recovery within established ERAS pathways.

Higher ASA scores are associated with increased post-operative complications and delayed recovery.^32^ Prehabilitation, incorporating physical training, nutritional support, and psychological preparation, can improve respiratory and overall health, potentially lowering surgical risk.^33^ Embedding a prehabilitation component into the ERAS pathway, as recommended by ERAS guidelines,^4^ may further enhance patient outcomes through structured pre-operative optimization.

### Limitations

This observational study is limited by the lack of a contemporaneous control group, randomization, and blinding, introducing potential for confounding and information bias. While the effects of ERAS elements cannot be definitely isolated, prior studies recognize their benefit.^20^^,^^28^ Without randomization, causality cannot be inferred. Patient-reported outcomes were not collected, precluding assessment from the patient’s perspective. Temporal changes in case-mix, including the increasing proportion of higher ASA grade patients, may have introduced residual confounding despite adjustment. Early mobilization may reflect rather than drive recovery; nonetheless, it remains a useful marker to identify at-risk patients.

## CONCLUSIONS

Our 10-year experience demonstrates that an ERAS programme for primary lung cancer resection is safe, effective, and sustainable. Its continued use was associated with improved short-term outcomes. Factors linked to shorter LOS included VATS, opioid-sparing analgesic techniques, and early mobilization. Conversely, increasing LOS was associated with older age, open/converted surgery, and ICU/HDU admission. Identifying and targeting these risk factors may enhance recovery and further optimize ERAS implementation.

## Supplementary Material

ivaf216_Supplementary_Data

## Data Availability

Data will be shared upon reasonable request to the corresponding author.
