# Supplementary material for: Temporal Trends in Outcomes and Predictors of Length of Stay Following Lung Cancer Resection Over 10 Years With Enhanced Recovery After Surgery
Source: Interdiscip Cardiovasc Thorac Surg. 2025 Oct 17;40(10):ivaf216. doi: 10.1093/icvts/ivaf216 (PMC12548365; doi:10.1093/icvts/ivaf216)
Supplement: ivaf216_Supplementary_Data [file ivaf216_supplementary_data.zip › Supplementary Material ERAS10 2.1 .docx]

**Temporal trends in outcomes and predictors of length of stay following lung cancer resection over 10 years with Enhanced Recovery After Surgery**

Supplementary Material

Table S1: Baseline characteristics, operative details and ERAS programme adherence

Table S2: Adherence to individual ERAS elements across year groups

Table S3: Factors associated with Length of Stay (continuous outcome, all patients)

Table S4: Predictors of prolonged LOS on univariable and multivariable analysis (binary outcome, Patients Without Morbidity)

Table S5: Predictors of prolonged LOS on univariable and multivariable analysis (binary outcome, all patients included)

Table S6: Predictors of morbidity on univariable and multivariable analysis

**Figure S1:** Median ERAS adherence scores with interquartile ranges across year groups

Table S1: Baseline characteristics, operative details and ERAS programme adherence

|  | **All,** N=2,192^1^ |  | **Normal LOS**, N = 1,122*^1^* | **Prolonged LOS**, N = 1,070*^1^* | **p-value***^2^* |
| --- | --- | --- | --- | --- | --- |
| Age | 70 (63, 75) |  | 69 (63, 75) | 71 (64, 76) | <0.001 |
| Male sex | 1,089 (49.7%) |  | 500 (44.6%) | 589 (55.0%) | 0.002 |
| Procedure: |  |  |  |  | <0.001 |
| Lobectomy | 1,327 (60.5%) |  | 649 (57.9%) | 678 (63.4%) |  |
| Wedge resection | 509 (23.2%) |  | 322 (28.7%) | 187 (17.5%) |  |
| Segmentectomy | 220 (10.0%) |  | 127 (11.3%) | 93 (8.7%) |  |
| Pneumonectomy | 76 (3.5%) |  | 11 (1.0%) | 65 (6.1%) |  |
| Bi-lobectomy | 60 (2.7%) |  | 13 (1.2%) | 47 (4.4%) |  |
| Approach: |  |  |  |  | <0.001 |
| VATS | 1,743 (79.5%) |  | 998 (89.0%) | 745 (69.6%) |  |
| Open | 316 (14.4%) |  | 89 (7.9%) | 227 (21.2%) |  |
| Converted | 133 (6.1%) |  | 35 (3.1%) | 98 (9.2%) |  |
| ASA: |  |  |  |  | <0.001 |
| 1 | 50 (2.3%) |  | 33 (2.9%) | 17 (1.5%) |  |
| 2 | 1,043 (47.5%) |  | 596 (53.1%) | 447 (41.8%) |  |
| 3 | 1,091 (49.7%) |  | 490 (43.6%) | 601 (56.2%) |  |
| 4 | 8 (0.4%) |  | 3 (0.3%) | 5 (0.5%) |  |
| Pre-operative Creatinine (umol/L) | 42 (30, 56) |  | 41 (29, 54) | 43 (30, 57) | 0.11 |
| Pre-operative Hb (g/L) | 55 (45, 64) |  | 57 (47, 64) | 53 (43, 63) | <0.001 |
| Pre-operative Visit | 2,146 (100%) |  | 1,099 (100%) | 1,047 (100%) | 1 |
| Pre-operative Assessment | 2,146 (100%) |  | 1,099 (100%) | 1,047 (100%) | 1 |
| Explanation of ERAS | 1,788 (81.5%) |  | 916 (87.0%) | 872 (85.9%) | 0.2 |
| Day of surgery admission | 2,072 (99.1%) |  | 1,053 (100.0%) | 1,019 (99.0%) | 0.1 |
| Pre-operative carbohydrate drink | 1,093 (55.0%) |  | 575 (56.9%) | 518 (53.3%) | 0.06 |
| Avoid sedation | 2,049 (99.0%) |  | 1,043 (99.0%) | 1,006 (99.0%) | 0.2 |
| Prophylactic antibiotics | 1,949 (93.0%) |  | 989 (94.0%) | 960 (93.0%) | 0.9 |
| Regional Anaesthesia | 2,052 (98.2%) |  | 1,031 (98.0%) | 1,021 (99.0%) | 0.002 |
| Warming | 2,070 (99.0%) |  | 1,051 (99.0%) | 1,019 (99.0%) | 0.3 |
| Avoidance of post-operative IV fluids | 2,079 (100%) |  | 1,055 (100%) | 1,024 (100%) | 0.12 |
| Avoidance of opioids | 82 (3.9%) |  | 48 (4.5%) | 34 (3.3%) | 0.14 |
| Early return to oral feeding | 1,766 (85.3%) |  | 903 (85.3%) | 863 (84.3%) | 0.3 |
| Targeted post-operative nausea and vomiting therapy | 2,015 (97.2%) |  | 1,021 (97.1%) | 994 (97.3%) | 0.8 |
| Mobilisation within 24 hours | 1,059 (50.7%) |  | 864 (76.9%) | 706 (66.0%) | >0.001 |
| Adherence to ERAS protocol | 12 (10, 13) |  | 12 (11, 13) | 11 (10, 12) | <0.001 |
| HDU/ICU Admission | 622 (28.4%) |  | 152 (13.6%) | 470 (43.9%) | <0.001 |
|  |  | *^1^* Median (IQR); n (%) | | | |
| *^2^* Wilcoxon rank sum test; Pearson's Chi-squared test; Fisher's exact test | | | | | |

Table S2: Adherence to individual ERAS elements across year groups

|  | **2013-2015** N = 534*^1^* | **2016-2017** N = 398*^1^* | **2018-2019** N = 485*^1^* | **2020-2021** N = 386*^1^* | **2022-2023** N = 389*^1^* |
| --- | --- | --- | --- | --- | --- |
| Pre-operative assessment | 534 (100%) | 397 (100%) | 482 (100%) | 383 (100%) | 387 (100%) |
| Pre-operative Visit | 534 (100%) | 397 (100%) | 482 (100%) | 383 (100%) | 387 (100%) |
| Explanation of ERAS | 466 (87%) | 322 (81%) | 315 (83%) | 324 (84%) | 361 (93%) |
| Day of surgery admission | 527 (99%) | 396 (99%) | 378 (100%) | 385 (100%) | 386 (99%) |
| Pre-operative carbohydrate drink | 385 (72%) | 141 (35%) | 162 (43%) | 223 (67%) | 182 (54%) |
| Prophylactic antibiotics | 507 (95%) | 371 (93%) | 327 (86%) | 364 (95%) | 380 (98%) |
| Avoid Sedation | 530 (99%) | 397 (100%) | 373 (99%) | 373 (99%) | 376 (97%) |
| Regional anaesthesia | 514 (96%) | 392 (98%) | 379 (100%) | 380 (98%) | 387 (99%) |
| Avoidance of opioids | 21 (3.9%) | 19 (4.8%) | 28 (7.4%) | 1 (0.3%) | 13 (3.4%) |
| Avoidance of IV fluids | 534 (100%) | 398 (100%) | 379 (100%) | 383 (99%) | 385 (99%) |
| Warming | 534 (100%) | 398 (100%) | 372 (98%) | 377 (98%) | 389 (100%) |
| Early returning to oral feeding | 480 (90%) | 363 (91%) | 346 (91%) | 285 (74%) | 292 (75%) |
| Early mobilisation | 346 (65%) | 298 (75%) | 283 (75%) | 78 (20%) | 54 (14%) |

Table S3: Factors associated with Length of Stay (continuous outcome, all patients)

|  | **Univariable Analysis** | | | **Multivariable Analysis** | | |
| --- | --- | --- | --- | --- | --- | --- |
|  | **β Coefficient**^1^ | **95% CI**^2^ | **p-value** | **β Coefficient**^1^ | **95% CI**^2^ | **p-value** |
| Age | 1.0 | 0.7, 1.9 | <0.001 | 0.05 | 0.03, 0.08 | <0.001 |
| Male sex | 0.02 | 0.01, 0.05 | 0.12 |  |  |  |
| ASA | 1.4 | 0.6, 2.4 | 0.002 | 0.44 | 0.19, 0.98 | 0.03 |
| Year of Surgery group | -1.2 | -2.2, -0.26 | 0.013 | -1.0 | -1.9, -0.13 | 0.025 |
| Procedure |  |  |  |  |  |  |
| Lobectomy | — | — |  |  |  |  |
| Bilobectomy | 1.4 | -0.42, 3.3 | 0.13 | -0.72 | -2.3, 0.88 | 0.4 |
| Pneumonectomy | 7.5 | 5.9, 9.2 | <0.001 | 2.5 | 0.97, 4.0 | 0.001 |
| Segmentectomy | -1.3 | -2.3, -0.29 | 0.012 | -0.66 | -1.6, 0.23 | 0.15 |
| Wedge | -1.3 | -2.0, -0.56 | <0.001 | -0.81 | -1.5, -0.16 | 0.015 |
| Approach |  |  |  |  |  |  |
| VATS | — | — |  |  |  |  |
| Open | 2.3 | 1.1, 3.6 | <0.001 | 0.64 | -0.43, 1.7 | 0.2 |
| Converted | 3.5 | 2.6, 4.3 | <0.001 | 0.98 | 0.16, 1.8 | 0.020 |
| Pre-operative creatinine | 0.00 | 0.00, 0.01 | 0.3 |  |  |  |
| Pre-operative haemoglobin | -0.20 | -0.42, 0.01 | 0.065 | 0.00 | -0.18, 0.18 | >0.9 |
| Explanation of ERAS | -0.16 | -0.89, 0.56 | 0.7 |  |  |  |
| Day of surgery admission | -0.18 | -0.90, 0.55 | 0.6 |  |  |  |
| Pre-operative carbohydrate drink | -3.9 | -6.5, -1.4 | 0.002 | -3.8 | -6.4, -1.2 | 0.004 |
| Avoid Sedation | 0.32 | -0.21, 0.85 | 0.2 |  |  |  |
| Prophylactic antibiotics | -0.45 | -1.5, 0.57 | 0.4 |  |  |  |
| Regional anaesthesia | 0.95 | -1.1, 3.0 | 0.4 |  |  |  |
| Warming | -1.6 | -4.7, 1.5 | 0.3 |  |  |  |
| Avoidance of IV fluids | -0.45 | -1.1, 0.19 | 0.2 |  |  |  |
| Avoidance of opioids | -3.4 | -6.1, -0.60 | 0.017 | -4.8 | -9.5, -0.15 | 0.043 |
| Early returning to oral feeding | -0.38 | -1.1, 0.38 | 0.3 |  |  |  |
| Targeted nausea and vomiting therapy | -0.11 | -1.6, 1.4 | 0.9 |  |  |  |
| Early mobilisation | -6.7 | -13, -0.78 | 0.027 | -1.5 | -2.1, -0.87 | <0.001 |
| HDU/ ICU admission | 5.0 | 4.4, 5.6 | <0.001 | 3.9 | 3.2, 4.6 | <0.001 |
| Surgeon identifier^3^ |  |  | 0.21 |  |  |  |
| ^1^β = regression coefficient from linear model. Positive values indicate longer stay; negative values indicate shorter stay. *^2^*CI = Confidence Interval. ^3^ Categorical variable with 7 levels. p-value from likelihood ratio test comparing model with and without surgeon identifier | | | | | | |

Table S4: Predictors of prolonged LOS on univariable and multivariable analysis (binary outcome, Patients Without Morbidity)

|  |  | | | **Univariable Analysis** | | | **Multivariable Analysis** | | |
| --- | --- | --- | --- | --- | --- | --- | --- | --- | --- |
|  | | **All,** N=1,210^1^ | **OR**^1^ | | **95% CI**^1^ | **p-value** | **OR**^1^ | **95% CI***^1^* | **p-value** |
| Age | | 69 (63, 75) | 1.02 | | 1.01-1.03 | <0.001 | 1.02 | 1.01-1.04 | 0.002 |
| Male sex | | 611 (50%) | 0.80 | | 0.65-1.12 | 0.15 |  |  |  |
| ASA | | 2 (1.3) | 1.57 | | 1.26-1.97 | <0.001 | 1.31 | 1.00-1.72 | 0.049 |
| Year of Surgery | |  |  | |  |  |  |  |  |
| 2013-2015 | | 308 (25%) | Reference | |  |  |  |  |  |
| 2016-2017 | | 174 (14%) | 0.88 | | 0.61-1.28 | 0.5 | 0.81 | 0.52-1.26 | 0.4 |
| 2018-2019 | | 254 (21%) | 0.63 | | 0.45-0.89 | 0.008 | 0.66 | 0.43-1.01 | 0.058 |
| 2020-2021 | | 242 (20%) | 0.60 | | 0.43-0.85 | 0.004 | 0.58 | 0.37-0.89 | 0.014 |
| 2022-2023 | | 243 (20%) | 0.64 | | 0.46-0.90 | 0.010 | 0.77 | 0.49-0.99 | 0.04 |
| Procedure | |  |  | |  |  |  |  |  |
| Lobectomy | | 745 (61%) | — | | — |  |  |  |  |
| Bilobectomy | | 28 (2.3%) | 3.02 | | 1.33-7.76 | 0.012 | 0.99 | 0.39-2.75 | >0.9 |
| Pneumonectomy | | 38 (3.1%) | 5.38 | | 2.38-14.4 | <0.001 | 1.83 | 0.62-6.83 | 0.3 |
| Segmentectomy | | 122 (10%) | 0.49 | | 0.33-0.73 | <0.001 | 0.63 | 0.40-1.00 | 0.051 |
| Wedge | | 288 (24%) | 0.60 | | 0.45-0.79 | <0.001 | 0.62 | 0.44-0.86 | 0.005 |
| Approach | |  |  | |  |  |  |  |  |
| VATS | | 987 (81%) | — | | — |  |  |  |  |
| Open | | 158 (13%) | 3.86 | | 2.33-6.62 | <0.001 | 2.58 | 1.62-5.29 | <0.001 |
| Converted | | 76 (6.2%) | 4.21 | | 2.91-6.20 | <0.001 | 2.88 | 1.62-4.15 | <0.001 |
| Pre-operative creatinine | | 75 (64, 89) | 1.00 | | 1.00-1.01 | 0.14 |  |  |  |
| Pre-operative haemoglobin | | 136 (126, 145) | 0.90 | | 0.83-0.97 | 0.007 | 0.97 | 0.89-1.07 | 0.6 |
| Explanation of ERAS | | 999 (83%) | 0.78 | | 0.55-1.08 | 0.14 |  |  |  |
| Day of surgery admission | | 1,152 (95%) | 0.35 | | 0.05-1.64 | 0.2 |  |  |  |
| Pre-operative carbohydrate drink | | 616 (51%) | 0.67 | | 0.61-0.97 | 0.04 | 0.94 | 0.79-0.97 | 0.04 |
| Avoid Sedation | | 1,134 (94%) | 0.66 | | 0.22-1.90 | 0.4 |  |  |  |
| Prophylactic antibiotics | | 1,084 (90%) | 0.74 | | 0.46-1.18 | 0.2 |  |  |  |
| Regional anaesthesia | | 1,141 (94%) | 1.06 | | 0.95-46.1 | 0.2 |  |  |  |
| Warming | | 1,148 (95%) | 0.50 | | 0.13-1.68 | 0.3 |  |  |  |
| Avoidance of IV fluids | | 1,155 (96%) | 0.44 | | 0.02-4.64 | 0.5 |  |  |  |
| Avoidance of opioids | | 51 (4.2%) | 0.65 | | 0.33-0.98 | 0.04 | 0.64 | 0.26-0.96 | 0.03 |
| Early returning to oral feeding | | 974 (80%) | 0.81 | | 0.59-1.11 | 0.2 |  |  |  |
| Targeted nausea and vomiting therapy | | 1,115 (92%) | 0.98 | | 0.52-1.86 | >0.9 |  |  |  |
| Early mobilisation | | 557 (46%) | 0.67 | | 0.57-0.93 | 0.01 | 0.84 | 0.62-0.96 | 0.03 |
| HDU/ ICU admission | | 350 (29%) | 4.62 | | 3.53-6.09 | <0.001 | 3.17 | 2.27-4.46 | <0.001 |
| Surgeon identifier^2^ | |  |  | |  | 0.067 |  |  |  |
|  | *^1^*OR=Odds Ratio, CI = Confidence Interval  *^2^* Categorical variable with 7 levels. p-value from likelihood ratio test comparing model with and without surgeon identifier. | | | | | | | | |

Table S5: Predictors of prolonged LOS on univariable and multivariable analysis (all patients included)

|  | | **Univariable Analysis** | | | **Multivariable Analysis** | | |
| --- | --- | --- | --- | --- | --- | --- | --- |
|  | **OR**^1^ | | **95% CI**^1^ | **p-value** | **OR**^1^ | **95% CI***^1^* | **p-value** |
| Age | 1.03 | | 1.01-1.05 | 0.001 | 1.04 | 1.02-1.06 | <0.001 |
| Male sex | 0.78 | | 0.53-1.15 | 0.20 |  |  |  |
| Year of Surgery |  | |  |  |  |  |  |
| 2013-2015 | Reference | |  |  |  |  |  |
| 2016-2017 | 1.04 | | 0.90-1.25 | 0.70 |  |  |  |
| 2018-2019 | 0.98 | | 0.92-1.13 | 0.43 |  |  |  |
| 2022-2021 | 0.93 | | 0.81-1.10 | 0.21 |  |  |  |
| 2022-2023 | 0.88 | | 0.80-1.05 | 0.07 | 0.97 | 0.85-1.11 | 0.65 |
| Procedure |  | |  |  |  |  |  |
| Lobectomy | Reference | |  |  |  |  |  |
| Bi-lobectomy | 1.13 | | 0.94-1.12 | 0.15 |  |  |  |
| Pneumonectomy | 6.12 | | 1.64-39.64 | 0.019 | 1.18 | 0.26-7.89 | 0.84 |
| Segmentectomy | 0.98 | | 0.84-1.11 | 0.32 |  |  |  |
| Wedge resection | 0.78 | | 0.61-0.95 | 0.03 | 0.92 | 0.87-0.98 | 0.045 |
| Approach |  | |  |  |  |  |  |
| VATS | Reference | |  |  |  |  |  |
| Converted | 1.39 | | 1.25-1.61 | <0.001 | 0.38 | 0.24-0.60 | <0.001 |
| Open | 1.15 | | 1.03-1.36 | 0.03 | 1.04 | 1.01-1.20 | 0.04 |
| Pre-operative creatinine | 1.01 | | 1.01-1.06 | 0.01 | 1.00 | 0.99-1.01 | 0.13 |
| Pre-operative haemoglobin | 1.00 | | 0.98-1.01 | 0.45 |  |  |  |
| Explanation of ERAS | 1.61 | | 0.84-3.19 | 0.16 |  |  |  |
| Day of surgery admission | 0.2 | | 0.01-1.27 | 0.15 |  |  |  |
| Pre-operative carbohydrate drink | 0.58 | | 0.37-0.89 | 0.013 | 0.83 | 0.70 - 0.97 | 0.04 |
| Prophylactic antibiotics | 0.55 | | 0.19-1.47 | 0.25 |  |  |  |
| Regional anaesthesia | 2.16 | | 0.97-5.14 | 0.15 |  |  |  |
| Avoidance of opioids | 1.05 | | 0.47-2.39 | 0.94 |  |  |  |
| Early returning to oral feeding | 0.74 | | 0.33-1.65 | 0.47 |  |  |  |
| Targeted post-operative nausea and vomiting therapy | 1.59 | | 0.70-3.73 | 0.27 |  |  |  |
| Early mobilisation | 0.26 | | 0.16 0.40 | <0.001 | 0.77 | 0.60 - 0.91 | 0.04 |
| HDU/ ICU admission | 4.95 | | 3.03-8.36 | <0.001 | 3.72 | 2.91 - 4.78 | <0.001 |
| *^1^*OR = Odds Ratio, CI = Confidence Interval | | | | | | | |

Table S6: Predictors of 30-day morbidity on univariable and multivariable analysis (all patients included)

|  | | **Univariable Analysis** | | | **Multivariable Analysis** | | |
| --- | --- | --- | --- | --- | --- | --- | --- |
|  | **OR**^1^ | | **95% CI**^1^ | **p-value** | **OR**^1^ | **95% CI***^1^* | **p-value** |
| Age | 1.07 | | 1.04-1.15 | 0.02 | 1.05 | 1.02, 1.16 | 0.03 |
| Male sex | 1.05 | | 0.86, 1.28 | 0.6 |  |  |  |
| Year of Surgery |  | |  |  |  |  |  |
| 2013-2015 | Reference | |  |  |  |  |  |
| 2016-2017 | 1.05 | | 0.67-1.31 | 0.56 |  |  |  |
| 2018-2019 | 1.09 | | 0.79-1.34 | 0.4 |  |  |  |
| 2022-2021 | 0.71 | | 0.51-0.98 | 0.039 | 1.04 | 1.00, 1.20 | 0.051 |
| 2022-2023 | 0.73 | | 0.53-1.00 | 0.054 | 0.94 | 0.81, 0.98 | 0.03 |
| Procedure |  | |  |  |  |  |  |
| Lobectomy | Reference | |  |  |  |  |  |
| Bi-lobectomy | 1.35 | | 0.76, 2.42 | 0.3 |  |  |  |
| Pneumonectomy | 3.11 | | 1.78-24.41 | 0.01 | 1.18 | 1.06-7.89 | 0.04 |
| Segmentectomy | 1.01 | | 0.74, 1.39 | 0.7 |  |  |  |
| Wedge resection | 0.78 | | 0.85-1.04 | 0.08 | 0.98 | 0.91-1.16 | 0.42 |
| Approach |  | |  |  |  |  |  |
| VATS | Reference | |  |  |  |  |  |
| Open | 1.09 | | 1.02-2.61 | 0.035 | 1.02 | 1.01-1.60 | 0.05 |
| Converted | 1.84 | | 1.23-2.36 | 0.001 | 1.32 | 1.09, 1.76 | 0.01 |
| Pre-operative creatinine | 1.09 | | 0.95-1.76 | 0.31 |  |  |  |
| Pre-operative haemoglobin | 0.99 | | 0.92-1.41 | 0.56 |  |  |  |
| Explanation of ERAS | 0.94 | | 0.73, 1.22 | 0.7 |  |  |  |
| Day of surgery admission | 0.83 | | 0.28, 2.46 | 0.7 |  |  |  |
| Pre-operative carbohydrate drink | 0.95 | | 0.79, 1.14 | 0.5 |  |  |  |
| Avoid pre-operative sedation | 1.36 | | 0.54, 3.70 | 0.5 |  |  |  |
| Prophylactic antibiotics | 0.97 | | 0.67-0.99 | 0.05 | 0.89 | 0.89-1.05 | 0.08 |
| Regional anaesthesia | 0.91 | | 0.44-1.89 | 0.8 |  |  |  |
| Warming | 2.48 | | 0.73-11.3 | 0.2 |  |  |  |
| Avoidance of opioids | 0.81 | | 0.50-1.30 | 0.4 |  |  |  |
| Early returning to oral feeding | 0.99 | | 0.75-1.29 | >0.9 |  |  |  |
| Targeted post-operative nausea and vomiting therapy | 1.02 | | 0.61-1.75 | >0.9 |  |  |  |
| Early mobilisation | 0.56 | | 0.16-0.80 | <0.001 | 0.72 | 0.54-0.97 | 0.039 |
| HDU/ ICU admission | 4.95 | | 3.03-8.36 | <0.001 | 2.83 | 1.66-4.04 | 0.01 |
| *^1^*OR = Odds Ratio, CI = Confidence Interval | | | | | | | |


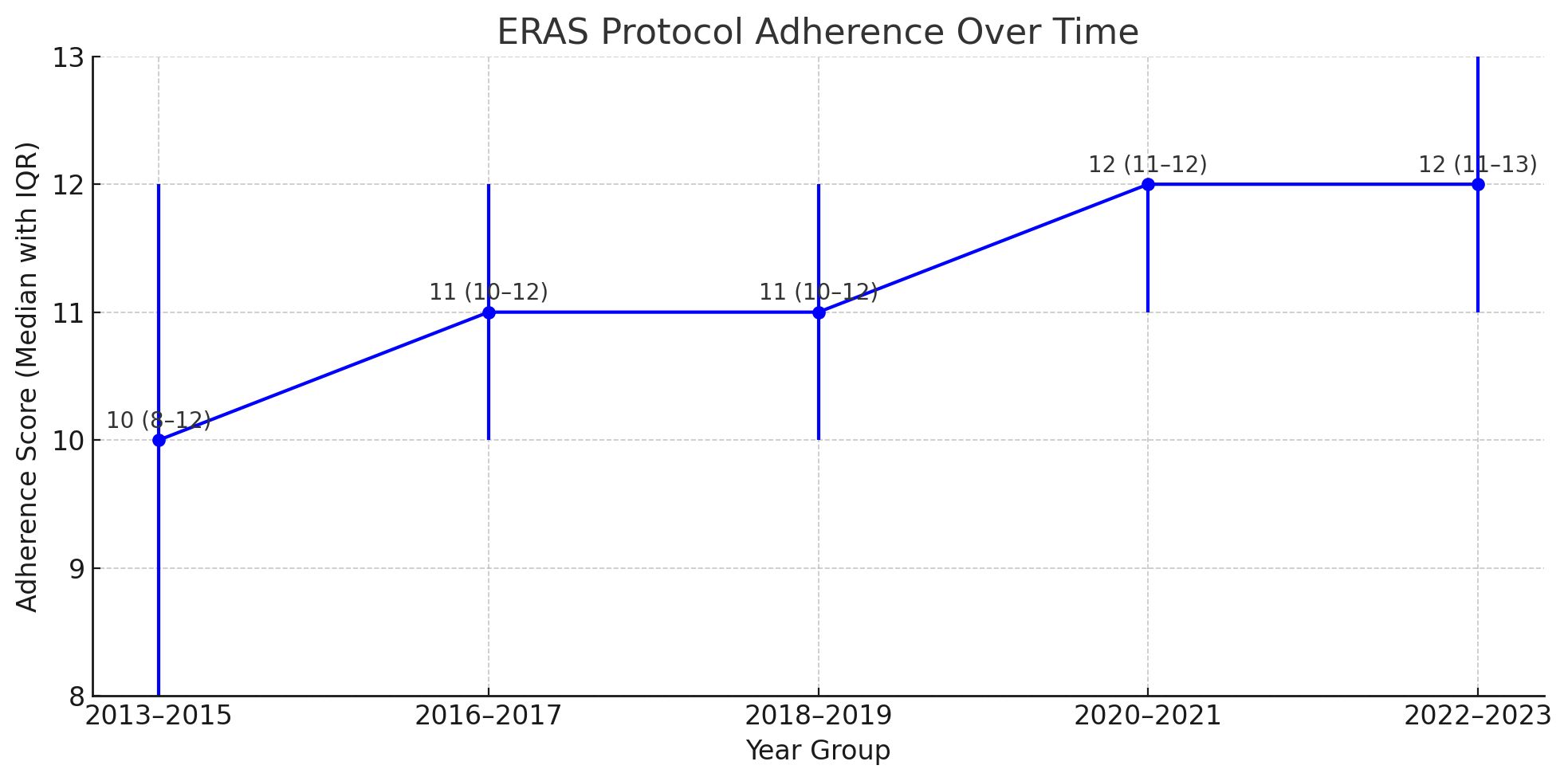


Figure S1: Median ERAS adherence scores with interquartile ranges across year groups
